# Maternal Thyroid Disorders and Their Effects on the Metabolic Profile of Breast Milk: A Systematic Review

**DOI:** 10.7759/cureus.103353

**Published:** 2026-02-10

**Authors:** Anna Kontogeorgou, Nikolaos Taprantzis, Marianna Politou, Theodoros Xanthos, George Kaparos, Theodora Boutsikou, Panagiotis Zoumpoulakis, Nicoletta Iacovidou, Dimosthenis Chrysikos, Theodore Troupis

**Affiliations:** 1 First Division of Pediatrics, "Aghia Sophia" Children's Hospital, National and Kapodistrian University of Athens, Athens, GRC; 2 Department of Anatomy, Medical School of National and Kapodistrian University of Athens, Athens, GRC; 3 Hematology Laboratory - Blood Bank, Aretaieio Hospital, Medical School of National and Kapodistrian University of Athens, Athens, GRC; 4 Department of Medicine, School of Health Sciences, University of West Attica, Athens, GRC; 5 Department of Biopathology, Aretaieio Hospital, Medical School of National and Kapodistrian University of Athens, Athens, GRC; 6 Department of Neonatology, Aretaieio Hospital, Medical School of National and Kapodistrian University of Athens, Athens, GRC; 7 Department of Food Science and Technology, University of West Attica, Athens, GRC

**Keywords:** human breast milk, human milk analyses, pregnant females, thyroid disorder, thyroid profile

## Abstract

Human breast milk composition is inextricably linked to maternal physiology, yet the impact of thyroid dysfunction on this biological matrix remains undercharacterized. Given that lactation serves as the primary metabolic conduit for the neonate, alterations in the milk metabolic profile could have profound developmental consequences. This study investigates metabolomic and proteomic differentiations in breast milk associated with maternal thyroid abnormalities to evaluate their potential implications for infant growth and neurodevelopment. A systematic search was conducted across PubMed, Embase, Web of Science, and Scopus to identify studies investigating the impact of maternal thyroid disorders on the metabolomic and proteomic composition of human breast milk. The Newcastle-Ottawa Scale was utilized to assess the risk of bias in the selected studies. The study was also registered in the International Prospective Register of Systematic Reviews (ID: CRD420261296307).

Nine studies were included in the review. The most consistent finding was a significant alteration of the milk lipidome, characterized by an increase in saturated fatty acids and a depletion of neurocritical lipids, such as glycerophospholipids and nervonic acid. Hypothyroidism was associated with reduced total protein content and downregulation of membrane proteins, including adipophilin and butyrophilin. Additionally, thyroid dysfunction correlated with decreased levels of sialylated oligosaccharides. Conversely, specific immune modulators, such as the Ig gamma-3 chain, were upregulated. Although the primary complement system components remained unchanged, a significant increase was observed in CD59, an inhibitor of complement activation.

Maternal thyroid dysfunction induces profound metabolomic and proteomic alterations in breast milk, fundamentally compromising its nutritional and structural integrity. The specific depletion of neurocritical lipids and immunomodulatory oligosaccharides, along with enzymatic downregulation, suggests a mechanism underlying impaired neurodevelopment, immune susceptibility, and digestive dysfunction in the neonate. These compositional defects may drive adverse "metabolic programming," predisposing offspring to immediate complications and long-term risks, including cognitive deficits and metabolic disorders. Consequently, strict management of maternal thyroid health is essential to preserve the biological quality of breast milk and optimize neonatal outcomes.

## Introduction and background

Human breast milk is unequivocally established as the optimal nutritional source for the neonate, providing a complex matrix of vital nutrients, immunological factors, and bioactive molecules (biological regulatory compounds) essential for the developing organism [1]. However, the quality of this biological supply is inextricably linked to maternal physiology; various systemic health factors can significantly alter milk composition and, by extension, affect the growth and developmental trajectory of the infant [2]. Thyroid dysfunction represents one of the most prevalent endocrine disorders among pregnant and lactating women, capable of exerting profound effects on both maternal homeostasis (state of internal balance) and neonatal outcomes. Given that lactation serves as the primary metabolic conduit between mother and child, understanding how thyroid pathology influences the breast milk metabolome, the complete set of small-molecule chemicals found within the milk, and structural integrity is a critical area of investigation [2,3]. Consequently, an increasing body of research has turned to advanced metabolic composition analyses to elucidate the detailed mechanistic links between maternal health factors and neonatal development.

The utility of this approach is well supported by the literature. Previous studies involving both animal models and human cohorts [4-7] have successfully demonstrated clear divergences in the plasma metabolic profiles of mothers with fetal growth disorders compared with healthy controls, changes which are frequently mirrored in the offspring. These findings establish metabolomics (the study of chemical processes involving metabolites) and proteomics (large-scale study of proteins) as a highly promising methodology for investigating the correlation between maternal metabolic disturbances and the subsequent development of neonatal pathology. This is primarily achieved by precisely identifying altered metabolites and disrupted metabolic pathways [8-11].

Building upon this framework, the present study is premised on the hypothesis that the metabolic footprint of human breast milk is significantly modified by the presence of maternal thyroid hormone disturbances. To this end, this study aims to comprehensively investigate the differentiations in the metabolomic and proteomic composition of breast milk from mothers with thyroid abnormalities and to critically evaluate the potential impact of these alterations on infant growth, neurodevelopment, and long-term physiological health.

## Review

Material and methods

We systematically searched through the PubMed, Embase, Web of Science, and Scopus databases. The search was conducted between September 2025 and December 2025 and covered the period from each database's inception to December 2025. The keywords that were used: (“thyroid disease” OR “hypothyroidism” OR “hyperthyroidism” OR “thyroid dysfunction”) AND (“human milk” OR “breast milk” OR “colostrum” OR “lactation”) AND (“metabolome” OR “proteome” OR “lipidome” OR “metabolic profile” OR “composition” OR “infant development”), in order to collect studies and information that would fit the criteria of our review. This analysis was also registered in the International Prospective Register of Systematic Reviews (ID: CRD420261296307). Since this paper used only published data, any Institutional Review Board approval or any other consent was not needed. Our search was limited to studies conducted in English, while additional data were obtained by screening the references of other articles. We conducted a systematic review with narrative synthesis, as the heterogeneous nature of the data precluded a quantitative meta-analysis. Specifically, the majority of included studies lacked standardized numerical reporting or utilized qualitative methodologies, preventing the statistical pooling of results.

The final set of studies and articles was decided based on the predefined selection of criteria, which included: 1) studies that reported the impact of thyroid diseases in pregnant women on the metabolomic composition of breast milk and 2) studies that showed the association between thyroid hormonal discrepancy and any biochemical content of human milk (e.g., proteins). The exclusion criteria include the following: 1) studies that were based on animals and not humans, 2) studies that did not investigate the impact of thyroid hormones, and 3) studies that did not study the metabolic profile of human breast milk. Finally, this review did not include abstracts from conferences or any study that did not report the data sufficiently. In terms of risk of bias assessment, the Newcastle-Ottawa Scale risk of bias assessment tool was utilized to evaluate each of the included studies (Table 1) [12].

**Table 1:** Risk of bias assessment based on the Newcastle-Ottawa Scale

Study	Selection	Comparability	Outcome	Overall
Fotakis et al. [13]	4/4	1/2	3/3	Good
Tyson et al. [14]	4/4	2/2	3/3	Good
Kivinen et al. [15]	2/4	1/2	3/3	Fair
Motil et al. [16]	3/4	1/2	2/3	Fair
Chen et al. [17]	3/4	1/2	3/3	Good
Jin et al. [18]	3/4	1/2	3/3	Good
Arias-Borrego et al. [19]	3/4	1/2	3/3	Good
Biddulph et al. [20]	3/4	2/2	3/3	Good
Altinoz et al. [21]	4/4	1/2	2/3	Good

Results

Search Strategy and Selection

**Figure 1:**
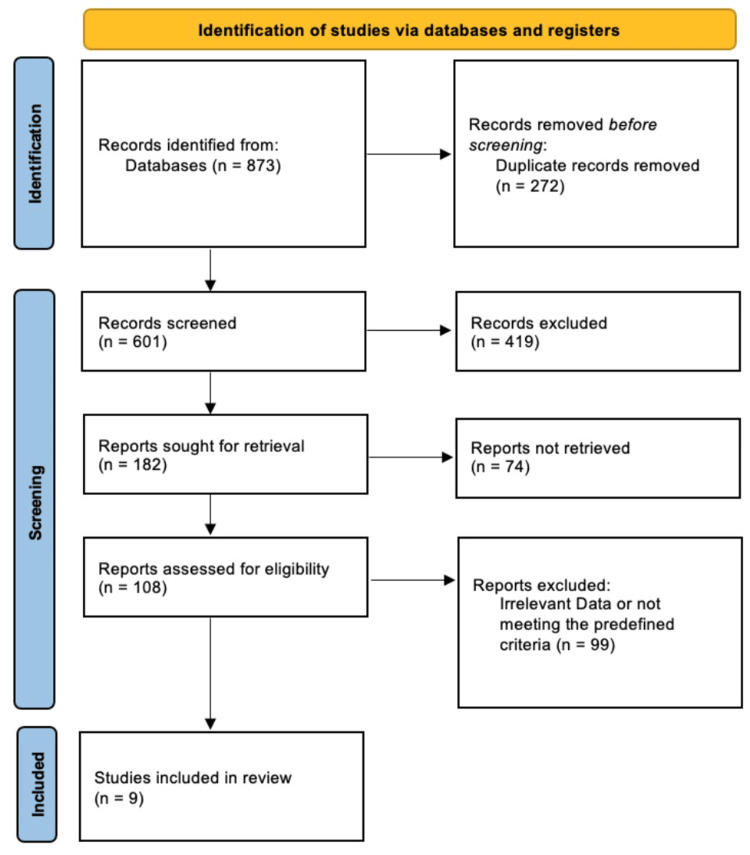
PRISMA flowchart PRISMA: Preferred Reporting Items for Systematic reviews and Meta-Analyses

Our systematic search initially identified 873 records from the target databases. Following the removal of duplicates and the screening of titles/abstracts against our exclusion criteria, 108 full-text articles were assessed for eligibility. Ultimately, nine studies met the strict inclusion criteria and were selected for this review (Figure 1).

Study Characteristics

The included studies covered a diverse range of thyroid dysfunctions affecting the maternal population. Specifically, five studies assessed mothers with hypothyroidism, two investigated increased thyrotropin-releasing hormone levels, one focused on iodine deficiency, and one examined varied thyroid hormone levels. The study characteristics and the results from the milk composition analyses are presented in Tables 2, 3.

**Table 2:** Study characteristics GC-MS: gas chromatography coupled to mass spectrometry

Study	Number of mothers	Assessment method for milk composition	Risk of bias assessment
Fotakis et al. [13]	90	MetaboAnalyst 5.0 (University of Alberta, Edmonton, AB), nuclear magnetic resonance spectroscopy	Low risk
Tyson et al. [14]	30	Infrared milk analyzer	Low risk
Kivinen et al. [15]	12	Gas-liquid chromatography	Moderate risk
Motil et al. [16]	12	Micro-Kjeldahl method adiabatic bomb calorimetry	Moderate risk
Chen et al. [17]	18	High-resolution mass spectrometry and liquid chromatography mass	Low risk
Jin et al. [18]	60	Bicinchoninic acid protein assay kit, ColorMixed protein marker	Low risk
Arias-Borrego et al. [19]	40	Inductively coupled plasma with triple quadrupole mass spectrometry, GC-MS, ultra-high performance liquid chromatography with quadrupole time-of-flight mass spectrometry	Low risk
Biddulph et al. [20]	101	Spectrophotometric enzymatic assay, bicinchoninic acid assay, modified creamatocrit method	Low risk
Altinoz et al. [21]	78	GC-MS system	Low risk

**Table 3:** Effects of maternal thyroid hormones on the metabolomic composition of milk LDL: low-density lipoprotein; VLDL: very-low-density lipoprotein; TRH: thyrotropin-releasing hormone; XDH: xanthine dehydrogenase; XO: xanthine oxidase; PASIII: periodic acid-Schiff III; PASIV: periodic acid-Schiff IV; TCA: tricarboxylic acid; SIDS: sudden infant death syndrome; HMOs: human milk oligosaccharides; TSH: thyroid-stimulating hormone; FT4: free thyroxine; SCD1: sudden cardiac death 1

Study	Type of thyroid hormone alteration	Effect on human milk metabolome	Clinical notes
Fotakis et al. [13]	Hypothyroidism	↑ Increased: threonine, lactate, choline, phosphocholine, glycerophosphocholine, LDL/VLDL; No significant change: lactose, alanine, citric acid, formic acid	Biomarker identification: These metabolites were identified as the most accurate markers for distinguishing thyroid disorder. Long-term risk: The specific metabolic patterns observed were theoretically linked to pathways involved in Alzheimer’s disease and schizophrenia, suggesting potential long-term neurocognitive risks for both mother and child
Tyson et al. [14]	Increased TRH	No significant change: total protein percentage, milk fat percentage	Clinical stability: Short-term TRH administration does not appear to alter macronutrient composition, nor does it affect infant weight or infant TSH levels
Kivinen et al. [15]	Increased TRH	No significant change: fatty acid content	Chronic TRH administration for stimulation should be cautiously monitored in the mother and baby
Motil et al. [16]	Multiple thyroid levels	↑ Increased: leucine incorporation (marker of protein synthesis) / ↑ Increased: urinary 3-methylhistidine (marker of muscle turnover)	Metabolic regulation: Nonessential amino acids (like proline and glutamate-glutamine) can become "limiting" during lactation if hormonal regulation is off
Chen et al. [17]	Hypothyroidism	↓ Decreased: metabolic proteins (alpha-enolase, fructose-1,6-bisphosphatase 1)/↓ Decreased: structural proteins (actin, tubulin)/↑ Increased: immune-related proteins (Ig gamma-3 chain, Ig kappa chain)/↑ Increased: CD59 (complement inhibitor)	No significant differences in weight or body length between the two groups
Jin et al. [18]	Hypothyroidism	↓ Decreased: total fat (1.34-fold)/↓ Decreased: total protein (1.13-fold)/↓ Decreased: milk fat globule membrane proteins (XDH/XO, PASIII, PASIV, butyrophilin, adipophilin)/no significant change: lactose	Immunological and growth risks: Reduced butyrophilin (critical for T-cell regulation) and lactadherin (essential for clearing apoptotic cells) may compromise the infant's immune homeostasis and negatively affect healthy growth
Arias-Borrego et al. [19]	Iodine-deficiency	↓ Decreased: glycerophospholipids (phosphatidylcholine, phosphatidylethanolamine, phosphatidylinositol)/↓ Decreased: TCA, urea cycle, and glycolysis intermediates/↑ Increased: saturated fatty acids (palmitic acid 1.62-fold, stearic acid 1.80-fold)/↑ Increased: triterpenoid ganoderic acid	Neurodevelopment: Deficiency in glycerophospholipids (neuronal membrane components) may impair cognitive function/Sudden infant death syndrome risk: Disrupted urea cycle metabolism has been theoretically linked to SIDS
Biddulph et al. [20]	Hypothyroidism	↓ Decreased: lacto-N-tetraose/↓ Decreased: sialylated human milk oligosaccharides (HMOs) (6'SL, LSTa, LSTb, LSTc)	Higher fat and higher energy milk content were observed in infants born with a low weight
Altinoz et al. [21]	Hypothyroidism	No significant change: total fat/↑ Increased: lignoceric acid (C24:0) (positively correlated with TSH, negatively with FT4)/↓ Decreased: SCD1 enzyme activity (defective conversion of saturated → unsaturated fats)	Impact on growth: 1. Negative: high stearic acid levels were significantly correlated with lower birth weight. 2. Positive: oleic, nervonic, gondoic, and behenic acids were positively correlated with birth weight

Impact on the Lipid Profile

The most consistent finding across the included studies was the significant modification of the milk lipidome. Thyroid-deficient states were characterized by a marked shift in fatty acid saturation. Multiple studies reported an accumulation of saturated fatty acids, with significant increases in stearic acid (C18:0) (1.80-fold) and palmitic acid (C16:0) (1.62-fold) [19,21]. Concurrently, lipids critical for neural structure were notably depleted. Iodine deficiency was associated with significantly reduced levels of glycerophospholipids [19], while the concentrations of nervonic acid and lignoceric acid showed direct statistically significant correlations with maternal thyroid-stimulating hormone and free thyroxine levels [21].

Impact on the Proteome and Glycome

Beyond lipids, thyroid status appeared to regulate the abundance of specific proteins and oligosaccharides. In terms of protein metabolism, hypothyroidism was linked to a quantifiable reduction in total protein content (approximately 1.13-fold lower in the disease group) and a significant downregulation of specific membrane-bound proteins, including adipophilin and butyrophilin [18]. Regarding complex sugars, Biddulph et al. observed a significant negative association between maternal thyroid dysfunction and the concentration of sialylated human milk oligosaccharides (HMOs), specifically noting decreased levels of LSTa, LSTb, and 6'SL [20].

Immunological Modifications

Distinct from metabolic substrates, alterations in immunological modulators were also observed. Specifically, immunoglobulins, such as the Ig gamma-3 chain C region and the Ig kappa chain V-I region, were significantly upregulated in hypothyroid mothers [17]. In contrast, the main components of the complement system remained largely unchanged between the groups, with the exception of CD59 (a complement inhibitor), which was significantly increased [17].

Discussion

Breast milk is rightly characterized as the essential source of nutrients, immunological modulators, and structural molecules required for neonatal development. While our systematic review indicates that maternal thyroid dysfunction does not consistently result in significant differences in infant birth weight or size, this absence of gross morphological change should not imply a lack of physiological impact. On the contrary, the altered composition of breast milk likely plays a deeper, more specific role in "metabolic programming." As milk is the neonate’s exclusive source of nutrition, any metabolomic deficiency or defect can have direct, cascading consequences on the function and maturation of critical biological systems [2,4].

Digestive Abnormalities

Maternal thyroid dysfunction appears to fundamentally degrade the caloric and structural quality of breast milk, creating an immediate challenge for infant energy acquisition. Research indicates that milk from hypothyroid mothers is metabolically deficient, characterized by significant reductions in total protein (1.13-fold) and fat (1.34-fold) content [18]. However, the deficit extends beyond simple macronutrient density.

Proteomic analysis reveals a uniform downregulation of key metabolic enzymes in colostrum, specifically glyceraldehyde-3-phosphate dehydrogenase, Enolase 1, and lactate dehydrogenase B. This suggests the milk lacks the catalytic support necessary to assist the infant’s immature gastrointestinal tract [17,22]. This enzymatic deficiency is compounded by structural alterations in the milk emulsion; hypothyroid milk exhibits significantly smaller milk fat globules and altered rheology (viscosity), which may impair oral processing and lipid absorption efficiency [18]. Clinically, this metabolic burden correlates with observed growth retardation and a prolonged duration of neonatal jaundice (50 vs. 30 days in controls) [17].

Lipidomic and Neurodevelopmental Risks

Perhaps the most critical consequence of maternal thyroid dysfunction is the alteration of the lipidome, which serves as the primary substrate for the rapidly developing neonatal brain. Thyroid hormones are essential for the enzymes (such as SCD1 and elongases) that synthesize complex brain lipids. When maternal thyroid function is compromised, this synthesis fails, leading to a metabolic blockage [21,23].

Studies identify a marked depletion of glycerophospholipids (including phosphatidylcholine and phosphatidylinositol) and myelin-associated fatty acids (nervonic and lignoceric acid) in the milk of thyroid-deficient mothers [19,21]. As these lipids are the building blocks of neuronal membranes and white matter, their deficiency poses a theoretical threat to synaptic formation and myelination [19,24]. Network analyses suggest these early perturbations may establish the biological origins for neurocognitive deficits and increase susceptibility to psychiatric disorders, such as schizophrenia or Alzheimer’s disease, later in life [13,25].

Interestingly, the fetus appears to experience a "metabolic rescue" in this environment. In cases of maternal hypothyroxinemia, fetal growth loses its metabolic flexibility, as well as its thermoregulatory ability, and becomes statistically dependent on specific ω9-monounsaturated fatty acids (oleic, gondoic), which are key elements for the processes of adipogenesis, brown fat activation, and thermoregulation [21,26,27]. This suggests that, in the absence of adequate thyroxine, the infant relies heavily on these specific lipids to adapt to the extrauterine environment and avoid hypothermia.

Impairment of Innate Immunity

Thyroid dysfunction induces a complex shift in the immunological profile of breast milk, characterized by a trade-off between structural and active immunity. On the one hand, bioactive proteins such as butyrophilin (linked to T-cell regulation) and lactadherin (MFGE8) are reduced, potentially compromising immune regulation [18]. Additionally, the upregulation of the complement inhibitor CD59 may suppress the infant's innate capacity to resist pathogens [18].

Conversely, studies by Chen et al. and Arias-Borrego et al. report an upregulation of specific immune-related proteins, including Ig gamma-3 chain and triterpenoid ganoderic acid in the milk of hypothyroid women. This suggests a compensatory mechanism where the maternal system boosts antibody-mediated defense in response to metabolic stress [17,19].

However, this protection is potentially undermined by changes in the glycome. Maternal thyroid dysfunction is associated with reduced concentrations of sialylated HMOs, such as LSTa, LSTb, and 6'SL. Since these oligosaccharides function as soluble "decoy receptors" preventing pathogen adhesion in the gut, their depletion may leave the infant more vulnerable to infection [20,28]. Furthermore, as sialylated HMOs are a key source of sialic acid for the brain, their reduction impacts both immune defense and neurodevelopment simultaneously [20,28].

Clinical Pathologies and Long-Term Effects

The cumulative effect of these metabolomic alterations predisposes the offspring to specific pathologies. Beyond neurocognitive risks, the disruption of nitrogen metabolism, evidenced by significantly reduced urea and uric acid levels in the milk of iodine-deficient mothers, has been theoretically linked to the etiology of sudden infant death syndrome due to impaired nitrogen homeostasis [19,29]. Furthermore, the "fetal programming" effects of this altered metabolic exposure may extend into adulthood, potentially predisposing the offspring to metabolic diseases such as diabetes mellitus [13].

## Conclusions

This systematic review demonstrates a clear correlation between maternal thyroid abnormalities and significant alterations in the metabolic composition of human breast milk. These findings underscore the critical link between maternal health and physiological neonatal development. Thyroid dysfunction fundamentally compromises the nutritional quality of breast milk by inducing specific metabolomic and proteomic shifts. Consequently, neonates may be predisposed to various disorders due to the resulting deficiencies in key metabolites, structural molecules, and immunoregulators.
